# Combination of drug-eluting bead transarterial chemoembolization and PD-1 inhibitor for treatment of unresectable head and neck squamous cell carcinoma: an initial, short-term clinical experience in a single-center retrospective cohort study

**DOI:** 10.3389/fimmu.2025.1615440

**Published:** 2025-07-23

**Authors:** Bo Sun, Jin Qi Gao, Ruo Jie Li, Lei Song, Na Li, Fei Gao

**Affiliations:** ^1^ Department of Oral and Maxillofacial Surgery, The Second Hospital of Dalian Medical University, Dalian, Liaoning, China; ^2^ Department of Intervention, The Second Hospital of Dalian Medical University, Dalian, Liaoning, China; ^3^ Department of Medical Oncology, The Second Hospital of Dalian Medical University, Dalian, Liaoning, China

**Keywords:** camrelizumab, drug-eluting CalliSpheres^®^ -beads, head and neck malignancies, squamous cell carcinoma, transarterial chemoembolization

## Abstract

**Introduction:**

This study investigated the clinical efficacy and safety of CalliSpheres^®^ drug-eluting bead transarterial chemoembolization (DEB-TACE) combined with programmed death protein (PD)-1 inhibitors for treatment of unresectable head and neck squamous cell carcinoma (HNSCC).

**Methods:**

Clinical data of 31 patients with unresectable HNSCC were retrospectively analyzed. All patients received local treatment with DEB-TACE combined with systemic PD-1 inhibitor. Modified Response Evaluation Criteria in Solid Tumors (mRECIST) criteria were used to evaluate the tumor response at 1, 3 and 6 months after the first treatment. Progression-free survival and overall survival were recorded. The changes in quality of life before and after treatment and adverse reactions during treatment were recorded.

**Results:**

Patients were treated with DEB-TACE 51 (average 1.65 ± 0.51) times. At 1, 3 and 6 months after the first treatment, objective response rate was 96.77%, 87.09% and 74.19%, and disease control rate was 100%, 96.77% and 83.87%, respectively. As of October 31, 2024, the mean follow-up was 21.71 ± 9.56 months, median survival time was 9.0 months, and the median progression-free survival was 19.0 months. The adverse reactions related to DEB-TACE were mainly fever, pain, nausea and vomiting; all of which were mild and relieved after symptomatic treatment. Three patients had mild skin ulceration in the embolic area, which healed after symptomatic treatment, and no serious complications such as ectopic embolism occurred. The adverse reactions associated with PD-1 inhibitor treatment were mainly fatigue, hypothyroidism and rash. Most of these were grade 1/2, three patients had grade 3 adverse reactions, but no grade 4 adverse reactions occurred. One month after the first treatment, the scores of physical function, emotional function and general health status increased, and the scores of pain, insomnia and anorexia decreased, and quality of life was significantly improved.

**Discussion:**

Combination of DEB-TACE with PD-1 inhibitors is safe and effective for treatment of unresectable HNSCC, significantly improves quality of life, and warrants clinical promotion and application.

## 1 Introduction

Head and neck squamous cell carcinoma (HNSCC) is one of the most common malignant tumors. In 2022, there were >79,000 new cases and >40,000 deaths in China; far more than in other countries and the number is increasing annually (1). The insidious location of the lesion and lack of specific symptoms means that ~60% of tumors are locally advanced or advanced at the time of diagnosis, and ~10% have distant metastases (2). In general, patients with early-stage HNSCC have similar treatment outcomes with surgery or radiotherapy, and the 5-year survival rate is 70%–90% (3). However, the 5-year survival rate of patients with locally advanced HNSCC treated with concurrent chemoradiotherapy (CRT) is still not >60%, indicating that the current efficacy of CRT has not met the need for new treatment strategies (4, 5). The complex anatomical and physiological structure of the head and neck makes it important to explore effective clinical interventions to improve the prognosis of advanced or recurrent HNSCC that is no longer suitable for local surgery or radiotherapy.

Since the 1990s, platinum-containing dual-drug chemotherapy has become the standard first-line treatment for recurrent/metastatic HNSCC, but the positive effect on survival is limited, with a median of only 5.0–8.7 months (6). With the advancement of multidisciplinary integrated therapy and molecular targeted therapy, cetuximab combined with platinum drugs and 5-fluorouracil (5-FU) can extend median overall survival (OS) to 10.1 months, but the overall prognosis still needs to be improved (7, 8). In recent years, immune checkpoint inhibitors have brought major breakthroughs in the treatment of HNSCC, and the 5-year survival rate of patients has increased from 5.0% to 15.4%–23.9% (9). However, with the wide application of immune checkpoint inhibitors in clinical practice, the selection of immunotherapy regimens in different populations, evaluation of immunotherapy effects, and treatment of adverse reactions need to be continuously standardized and improved. In recent years, the development of interventional therapy has provided patients with more options. Our previous single-arm retrospective study confirmed the efficacy and safety of CalliSpheres^®^ drug-eluting bead transarterial chemoembolization (DEB-TACE) in the treatment of HNSCC. We observed necrotic changes in the tumor after treatment, and we have reason to believe that these changes may alter the local tumor environment. This combination with immunotherapy after surgery has a potential synergistic effect and promising initial efficacy in patients with unresectable HNSCC. The purpose of this study was to investigate the safety and efficacy of DEB-TACE combined with programmed death protein (PD)-1 inhibitor in the treatment of unresectable HNSCC.

## 2 Data and methods

### 2.1 Case selection

A retrospective cohort analysis was used to collect 31 cases with unresectable HNSCC treated with DEB-TACE combined with PD-1 inhibitors in the Second Hospital of Dalian Medical University from January 2020 to January 2024. The study was approved by the Hospital Ethics Committee and was conducted in accordance with the ethical standards set out in the 1964 Declaration of Helsinki and its subsequent amendments. Since our study was retrospective, written informed consent was waived by our Institutional Review Board. Inclusion criteria: (1) age 18–80 years, regardless of gender; (2) patients diagnosed with SCC or recurrent or metastatic HNSCC; (3) no active bleeding; (4) patients unable to undergo surgery or who did not respond to CRT; (5) refusal of surgery or chemoradiotherapy; (6) Eastern Cooperative Oncology Group score ≤2; and (7) at least 1 cycle of DEB-TACE+PD-1 inhibitor therapy. Exclusion criteria: (1) metastatic head and neck malignancies or other pathological types of tumors; (2) inability to effectively control distant metastatic lesions; and (3) lack of clinical and follow-up data.

### 2.2 Treatment methods

#### 2.2.1 Preparation of CalliSpheres^®^

DEBs of 100–300 μm (1 g/bottle, Suzhou Hengrui Galisheng Biomedicine Technology Co. Ltd., National Instrument injection standard 20153771072) were selected. A 20-ml syringe was used to extract the DEBs and normal saline; the DEBs were allowed to settle for 2–3 min in the syringe; and the supernatant was ejected from the syringe. Epirubicin (40 mg) was dissolved in 5 ml sterilized water for injection. The 20-ml syringe and 5-ml epirubicin syringe were connected with a three-way connection, and the epirubicin solution was slowly pushed into the syringe containing the DEBs. The syringe containing the DEBs and chemotherapeutic drugs was capped with a needle cap and shaken every 5 min for a total of 30 min. The DEBs carrying epirubicin were mixed with the non-ionic contrast agent iodixanol at a ratio of 1:1 and left for 5 min before use.

#### 2.2.2 DEB-TACE technology

The right femoral artery was punctured using the Seldinger method. A 5F arterial sheath was inserted and a hydrophilic membrane guidewire and 5F catheter were introduced. External carotid and subclavian arteriography was performed according to the lesion site to identify the blood supply artery. Superselective arterial intubation was performed, and the common supplying arteries included the superficial temporal, maxillary, mandibular, facial, sublingual and superior thyroid arteries. After angiography confirmed that there was no dangerous anastomosis, the tumor was embolized with the configured DEBs, and gelatin sponge particles were used as a supplementary embolization agent. The embolization endpoint was the loss of tumor staining on angiography.

#### 2.2.3 PD-1 inhibitor administration

PD-1 inhibitor was started within 3 days after the first application of TACE: 200 mg carrilizumab, 200 mg tirellizumab, 240 mg triplizumab or 200 mg pabolizumab intravenously, then every 3 weeks. Other adjuvant treatments were routinely given after surgery: anti-inflammatory, analgesic and antiemetic drugs and hydration.

### 2.3 Evaluation of therapeutic efficacy

All patients received enhanced computed tomography (CT)/magnetic resonance imaging every 1–3 months after the first treatment to observe the size of the lesion, degree of necrosis and whether there were new lesions. Based on the imaging results, short-term efficacy evaluation was performed with reference to the modified Response Evaluation Criteria in Solid Tumors (mRECIST). Complete response (CR): CT or magnetic resonance imaging showed loss of enhancement in the arterial phase of all target lesions. Partial response (PR): ≥30% reduction in the sum of the diameters of target lesions (arterial phase enhancement development). Progressive disease (PD): ≥20% increase in the sum of the diameters of target lesions (arterial phase enhancement development) or the development of new lesions. Stable disease (SD): a decrease in the sum of the diameters of the target lesions (arterial phase enhancement development) that did not achieve PR or an increase that did not reach PD. Objective response rate (ORR) was defined as the proportion of patients achieving CR and PR. Disease control rate (DCR) was defined as the proportion of patients achieving CR, PR and SD. Focus on observing the range of tumor activity, comprehensively evaluate the interventional effect and determine whether to receive interventional therapy again. Patients who achieved CR underwent clinical follow-up. If patients had PR, SD or PD after initial DEB-TACE, treatment was repeated after ensuring that the patient’s status returned to baseline. If patients still had PD after receiving two DEB-TACE sessions, they did not continue to receive this treatment method. Progression-free survival (PFS) and overall survival (OS) were recorded. OS was defined as the time from the start of the first intervention to death or last follow-up. PFS was defined as the time from the start of treatment to PD or death, or the time from the start of treatment to the last follow-up in patients who had not progressed or died.

### 2.4 Evaluation of quality of life and observation of adverse reactions

The European Organization for Research and Treatment of Cancer Quality of Life Core Questionnaire (version 3.0) was used for evaluation. The Questionnaire had a total of 30 items, including 5 functional subscales: somatic function, role function, cognitive function, emotional function and social function; 3 symptom subscales: fatigue, pain, and nausea and vomiting; one total quality of life subscale and 6 individual items: dyspnea, insomnia, loss of appetite, constipation, diarrhea and economic impact. Higher scores on the functional and overall quality subscales were better, and higher scores on the symptom subscales and single items were more severe. Adverse reactions were judged according to the National Cancer Institute Common Terminology Criteria for Adverse Events version 5.0, and the criteria were divided into grades 0–4.

### 2.5 Statistical analysis

SPSS version 20.0 was used for statistical analysis. The numerical data were represented as examples; normally distributed measurement data were represented by mean and standard deviation; and non-normally distributed data were represented by median (Q1, Q3). The χ^2^ test, paired *t* test and rank sum test were used for each type of data, respectively. The Kaplan–Meier method was used to calculate the survival time and plot the survival curve. *P*<0.05 was considered statistically significant.

## 3 Results

### 3.1 Baseline data of patients

**Figure 1:**
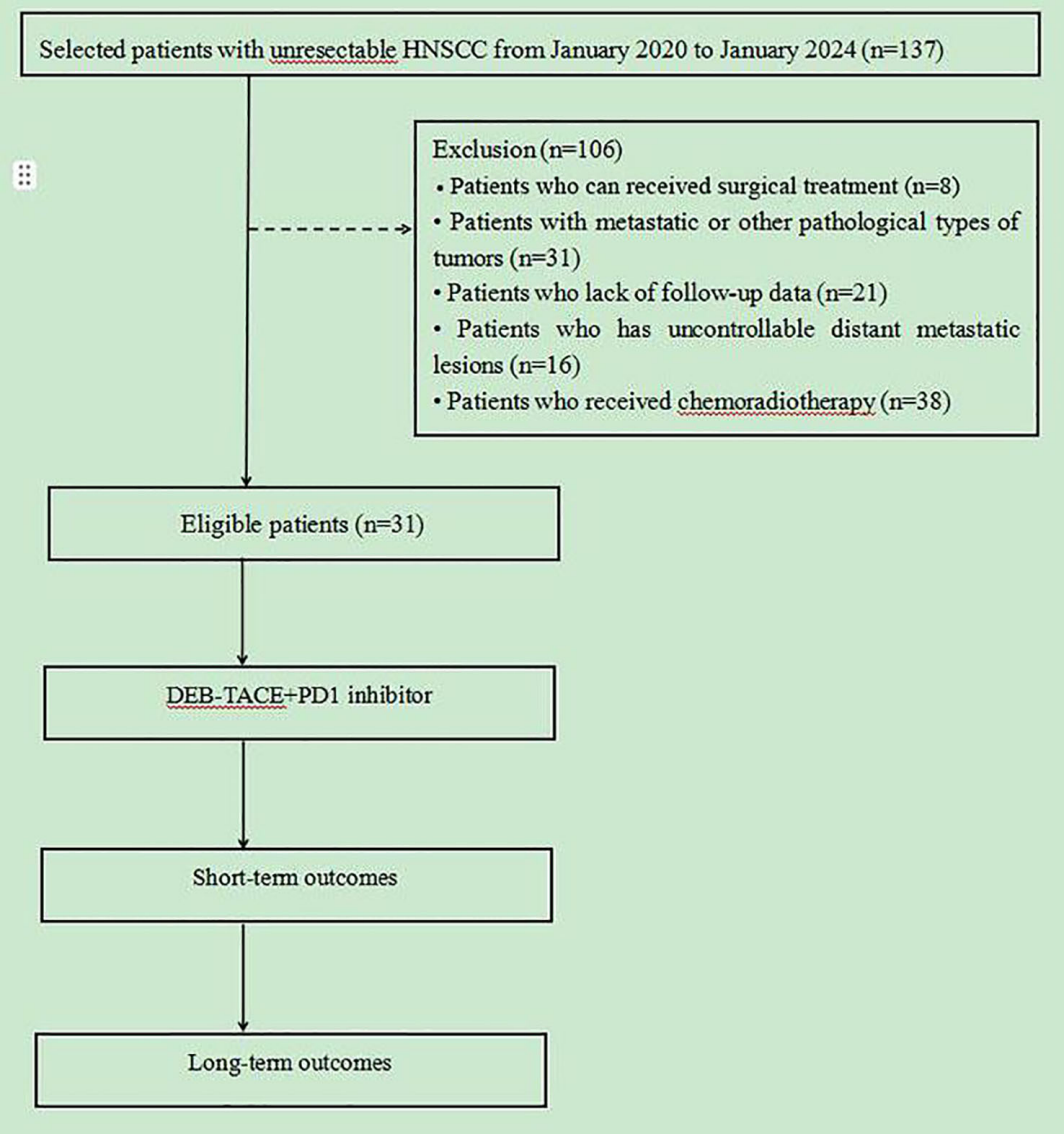
Flowchart of this study. 31 patients who underwent from January 2020 to January 2024 were collected, short- and long-term outcomes were compared.

From January 2020 to January 2024, 137 patients with HNSCC were admitted to the Cancer Center of the Second Hospital of Dalian Medical University were selected, and finally 31 patients were included for data analysis according to the inclusion and exclusion criteria, the entire process is shown in **Figure 1**. Among the 31 patients, the primary tumor sites included: the oral cavity (2 cases in the floor of the mouth, 1 in the oral cavity, 3 in the tongue, and 1 each in the gingiva and tonsils); pharynx (9 cases in the nasopharynx, 2 in the oropharynx and 5 in the hypopharynx); larynx (5 cases); and maxillary sinus and thyroid (1 case each). Interventional treatment was the first choice among 24 patients who had undergone surgery, CRT and other antitumor treatments (10 with active bleeding, 7 without previous treatment, 2 with hemorrhage, and 5 elderly/frail patients); patients in whom the tumor was surrounded locally by important blood vessels; and patients unable to undergo surgery and who refused CRT. The baseline data of the patients is shown in **Table 1**.

**Table 1:** Clinical features of 31 patients with unresectable HNSCC.

Characteristics	Enrolled Patients (n = 31)	%
Gender		
Male	18	58.06
Female	13	41.94
Age, mean ± SD	62.65 ± 11.85	
ECOG performance status		
0	6	19.35
1	22	70.97
2	3	9.68
Prior treatment		
Surgical	11	35.48
Chemoradiotherapy	24	77.42
Untreated	7	22.58
Combined bleeding		
Yes	10	32.26
No	21	67.74
Tumor size (cm), mean ± SD	5.36 ± 2.76	
Clinical stages		
Stage III	14	45.16
Stage IV	17	54.84
Frequency of DEB-TACE		
1	17	54.84
≥2	14	45.16

### 3.2 Short-term effect

**Figure 2:**
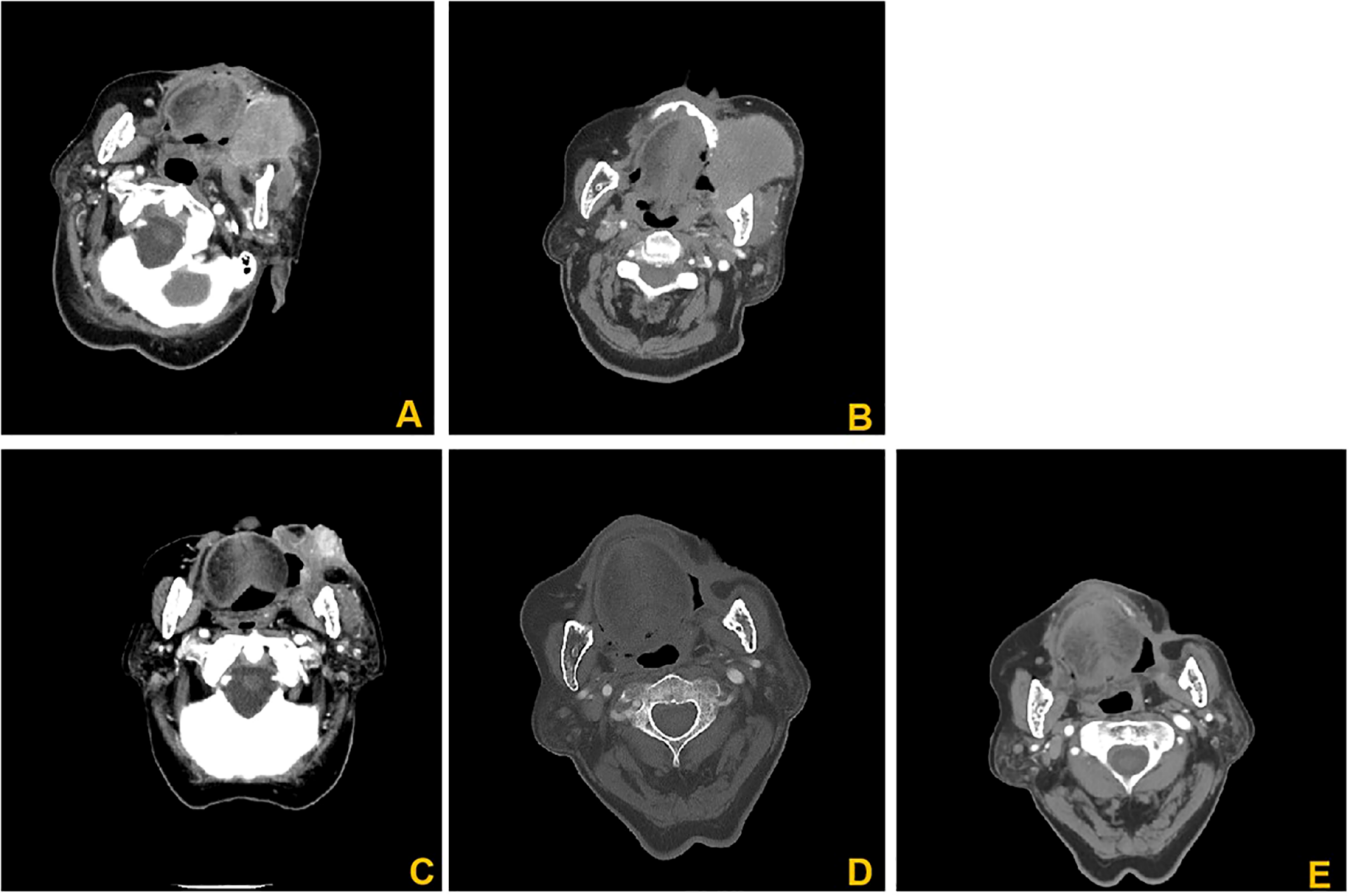
CT images of an 86-year-old female patients. **(A)** Enhanced CT images before interventional treatment; **(B)** Enhanced CT showed significant low-density changes of the tumor 7 days after treatment; **(C)** Enhanced CT showed that the lesion was significantly reduced, and no significant enhancement was found 1 months after treatment. **(D)** 3 months after treatment, the lesions basically disappeared; **(E)** 6 months after treatment, the features of the lesion were basically the same as those in D, without significant changes.

The 31 patients completed 51 DEB-TACE treatments, ranging from 1 to 3 times, with an average of 1.65 ± 0.51 times. Most of the patients showed good tumor necrosis by CT within 5–7 days after surgery. One month after the first treatment, all 31 patients were able to obtain imaging evaluation, 28 at 3 months after treatment and 26 at 6 months after treatment. According to the mRECIST criteria, at 1, 3 and 6 months after the first treatment, the ORR was 96.77%, 87.09% and 74.19%, and the DCR was 100%, 96.77% and 83.87%, respectively (**Table 2**). **Figure 2** presents the CT images of a 86-year-old female patient with stage II left cheek squamous cell carcinoma, and the patient was unfit for surgery, and received 4 cycles of chemotherapy and targeted therapy in the Department of Medical Oncology, yet the tumor progressed. Subsequently, the patients underwent DEB-TACE combined with camrelizumab (intravenous infusion every 3 weeks). One month post-procedure, the tumor significantly regressed. By three months post-procedure, the lesions nearly disappeared, and achieved complete remission (**Figure 2**).

**Table 2:** Short-term efficacy in 31 patients with unresectable HNSCC after DEB-TACE surgery [n, (%)].

Clinical response	1m (n=31)	%	3m (n=28)	%	6m (n=26)	%
CR	3	9.68	3	9.68	1	3.23
PR	27	87.09	24	77.41	22	70.96
SD	1	3.23	3	9.68	3	9.68
PD	0	0	1	3.23	5	16.13
ORR	30	96.77	27	87.09	23	74.19
DCR	31	100.00	30	96.77	25	83.87

### 3.3 Survival time and survival curve

**Figure 3:**
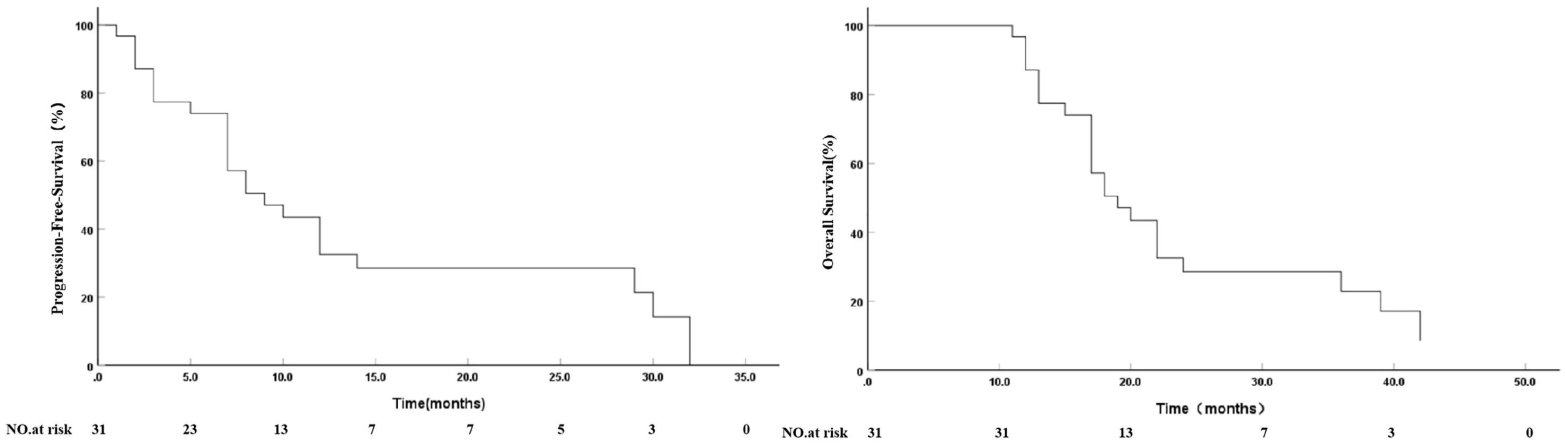
Kaplan-Meier analyses of progression-free survival and overall survival.

As of October 31, 2024, follow-up time was 11–42 months, with an average of 21.71 ± 9.56 months and median of 18 months. Survival analysis showed that median PFS was 9.0 months (95% confidence interval: 5.09–12.90 months) and 12-month PFS was 32.6%. Median OS was 19.0 months (95% confidence interval: 15.09–22.90 months) and 1-, 2- and 3-year cumulative survival rates were 87.1%, 28.5% and 22.8%, respectively (**Figure 3**).

### 3.4 Adverse reactions

The adverse reactions during DEB-TACE were mainly interventional embolic syndrome, such as fever, pain, nausea and vomiting, with the highest incidence of pain (n=22; 70.97%), followed by fever (n=15; 48.39%). Body temperature was 37.5–38.5°C, which was related to tumor necrosis and absorption, and was gradually relieved within 3–7 days after symptomatic treatment. Other adverse reactions were grade 1 or 2 and were generally relieved within 1 week after symptomatic treatment. Three (9.68%) patients had mild skin ulceration in the embolic area, which healed after symptomatic treatment, and no serious complications such as ectopic embolism occurred. The adverse reactions associated with PD-1 inhibitor treatment were mainly fatigue (n=11), hypothyroidism (n=6), rash (n=4), body surface capillary proliferation (n=3), hepatitis (n=3), pneumonia (n=2) and arthralgia (n=1). Most of these reactions were grade 1 or 2 and were relieved after symptomatic treatment and short suspension of medication. A small number of patients had grade 3 adverse reactions (including 2 cases of fatigue and 1 of rash), and no grade 4 adverse reactions occurred (**Table 3**).

**Table 3:** Incidence of Adverse Reactions of HNSCC patients during the treatment [Cases/(%)].

Events	Enrolled Patients (n = 31)	%
Nausea/vomiting	11	35.48
Pain	22	70.79
Fever	15	48.39
Fatigue	11	35.48
Rash	4	12.90
Capillary proliferation	3	9.68
Hypothyroidism	6	19.35
Pneumonia	3	9.38
Hepatitis	2	6.45
Arthralgia	2	6.45

### 3.5 Changes in quality of life

One month after the first treatment, the scores of physical function, emotional function and general health status increased; the scores of pain, insomnia and anorexia decreased; and quality of life was significantly improved (**Table 4**).

**Table 4:** Quality of life score M (Q1, Q3) of 31 patients before and after DEB-BACE treatment.

Item	Before treatment	1 month after treatment	Z value	P value
Physical function	86.7 (86.7, 93.3)	93.3 (93.3, 100)	3.331	0.016
Role function	66.7 (66.7, 83.3)	83.3 (66.7, 100)	0.879	0.164
Cognitive function	100 (83.3, 100)	100 (83.3, 100)	1.983	0.092
Emotional function	83.3 (67.7, 100)	100 (83.3, 100)	2.586	0.020
Social function	100 (91.7, 100)	100 (100,100)	0.259	0.369
General health status	66.7 (58.3, 68.7)	93.3 (93.3, 100)	3.291	0.017
Fatigue	11.1 (0, 22.2)	11.1 (0, 22.2)	4.651	0.010
Pain	33.3 (33.3, 50.0)	16.7 (16.7, 33.3)	2.058	0.087
Nausea and vomiting	0 (0, 33.3)	0 (0, 16.7)	2.365	0.053
Dyspnea	0 (0, 0)	0 (0, 0)	6.988	0.007
Insomnia	16.7 (16.7, 33.3)	0 (0, 16.7)	2.587	0.020
Anorexia	66.7 (66.7, 100)	16.7 (0, 66.7)	0.657	0.205
Constipation	16.7 (0, 33.3)	16.7 (0, 33.3)	0.298	0.301
Diarrhea	0 (0, 33.3)	0 (0, 33.3)	0.148	0.900
Economic impact	33.3 (33.3, 66.7)	33.3 (33.3, 66.7)	0.782	0.165

## 4 Discussion

Early HNSCC is mainly treated by surgery or radiotherapy, which may be combined with chemotherapy, targeting and other drug treatments (10–12). In recent years, the development of interventional therapy has provided patients with more options. Locally advanced HNSCC requires multimodal therapy. Surgery and radiotherapy combined with platinum-containing chemotherapy or anti-epidermal growth factor receptor targeted therapy and immunotherapy can achieve better outcomes (13). At present, cevenepam combined with concurrent CRT has achieved gratifying results in the treatment of patients with locally advanced HNSCC (14). Proton and heavy ion therapy, boron neutron capture therapy and magnetic-resonance-guided accelerator adaptive radiotherapy provide more treatment options for tumor patients (15–17).

However, clinical treatment of recurrent and refractory HNSCC may still be difficult. For patients with recurrent/metastatic HNSCC who are no longer suitable for local surgery or radiotherapy, the median OS of first-line chemotherapy alone (platinum+5-FU or platinum+paclitaxel) is 7.4–8.4 months. Compared with chemotherapy alone, cetuximab combined with platinum+5-FU significantly improved the response rate and long-term prognosis, with ORR increasing from 20%–27% to 36%–50%, median OS increasing from 7.4–8.4 to 10.1–10.2 months, and median PFS increasing from 3.3–4.2 to 5.5–5.6 months (8, 18). The high expression of PD ligand (PD-L)1 on the surface of HNSCC cells and the high inflammatory state of the immune microenvironment provide a theoretical basis for the application of PD-1 (19). The application of PD-1 monoclonal antibody improves treatment efficacy and prolongs OS. The CheckMate-141 study explored the second-line treatment of platinum-resistant patients with recurrent/metastatic HNSCC. In patients with relapsed HNSCC, nivolumab monotherapy significantly increased median OS (7.5 vs 5.1 months) and decreased incidence of grade 3/4 adverse events compared with systemic standard therapy (20). The KEYNOTE-048 study confirmed that, when comparing first-line therapy and the EXTREME regimen in patients with recurrent/metastatic HNSCC, pembrolizumab in combination with chemotherapy significantly improved response rates and long-term survival (21). The KEYNOTE-B10 study explored the efficacy and safety of pembrolizumab+carboplatin+paclitaxel in the first-line treatment of recurrent/metastatic HNSCC with a median follow-up of 8.2 months. The first 82 treated patients had an ORR of 43%, median DCR of 5.5 months, median PFS of 5.6 months, median OS of 12.1 months, 6-month OS rate of 73% and 12-month OS rate of 58% (22). In recent years, we have tried multiple regimens in combination with PD-1 monoclonal antibody for treatment of recurrent/metastatic HNSCC, which has achieved better therapeutic outcomes and a lower incidence of treatment-related adverse events (23, 24). Although the efficacy of PD-1 inhibitors in the treatment of advanced metastatic HNSCC has been confirmed, the occurrence of drug resistance cannot be avoided. In recurrent/metastatic HNSCC, high rates of primary or acquired resistance to PD-1 inhibitors have been reported due to the absence of antigenic proteins, defective antigen presentation, T cell failure/deletion, tumor insensitivity to T cells, presence of immunosuppressive cells, and/or presence of other suppressive immune checkpoints (25). In clinical practice, radiotherapy combined with immunotherapy can show a significant distant effect. Choi et al. reported two patients who received stereotactic body radiotherapy combined with immunotherapy for recurrent/metastatic HNSCC (26). The primary tumor shrank significantly and distant effects appeared after treatment, and no tumor recurrence was observed during follow-up. In this process, tumor cells undergo apoptosis after radiotherapy, and non-immunogenic cells are transformed into immunogenic cells to form an *in situ* vaccine. This activates T cells and kills tumor cells within and outside the radioactive field, while the combination of radiotherapy and immune checkpoint inhibitor showed a synergistic enhancement of the *in situ* vaccine effect.

TACE is one of the effective treatments for head and neck tumors. It can significantly increase the local drug concentration in tumors and reduce adverse reactions. In addition, it can embolize the tumor-supplying arteries, promote tumor necrosis and shrinkage, and improve overall treatment efficacy (27). Tumor necrosis can cause antigenic exposure, secondary to a series of immunological changes. At present, the commonly used embolic agents for TACE in head and neck malignancies include gelatin sponges, polyvinyl alcohol particles and embolic microspheres (28). Gelatin sponge microparticles have good elasticity and excellent delivery capacity, which significantly improve integration with target blood vessels, resulting in more complete embolization. However, due to their biodegradability, blood flow is mostly restored within a short period of time with high safety, but multiple interventions are required (29, 30). DEB-TACE is a novel interventional tumor therapy modality in which DEBs can permanently embolize tumor blood vessels and slowly release antitumor drugs in the tumor, thereby shrinking the tumor and controlling its development, so as to better reduce clinical symptoms and improve quality of life (31, 32). CalliSpheres microspheres are DEBs independently developed in China, which have been widely used in the interventional treatment of liver and lung cancers. In a retrospective study of DEB-TACE in the treatment of massive liver cancer, Zhao et al. found that CalliSpheres had several advantages over traditional DEBs in tumor control rate (33). However, there have been few studies of DEB-TACE in the treatment of head and neck malignancies. In our previous single-arm retrospective study, CalliSpheres-TACE was used to treat 15 patients with HNSCC, with a DCR of 100% at 1 month after surgery, DCR of 73.3% at 2 months after surgery, 1-year PFS rate of 34.1% and a 1-year OS rate of 38.9%. Although the study reported a small number of cases and inconsistent staging of enrolled patients, it further revealed the feasibility of using CalliSpheres for the treatment of head and neck malignancies (34).

In this study, TACE was performed using CalliSpheres to load epirubicin for the treatment of unresectable HNSCC, combined with PD-1 inhibitor therapy after surgery. ORR and DCR were 96.77% and 100% at 1 month after treatment, respectively, and remained at a high level of 74.19% and 83.87% at 6 months. We believe that it was mainly related to significant tumor necrosis after surgery. In our cases, re-examination at 1 month after surgery showed that the tumor had undergone a significant low-density necrotic change with uniform distribution, suggesting that the DEBs achieved effective embolization of the aneurysmal artery. In addition, their good drug-loading characteristics also played a role in tumor necrosis, and the interaction between the DEBs and chemotherapeutic drugs showed an enhancing effect. Follow-up survival analysis showed that the median PFS was 9.0 months and the 1-year PFS rate was 32.6%. Median OS was 19.0 months and the 1-, 2- and 3-year cumulative survival rates were 87.1%, 28.5% and 22.8%, respectively. We believe that the main reason for the better long-term efficacy was that the combination of interventional and immunological treatments improved the ORR. In our previous studies, DEB-TACE had a positive antitumor performance in patients with malignant tumors, with changes in the microenvironment including increased natural killer cells and CD4^+^/CD8^+^ ratios, and decreased levels of T regulatory cells and interleukin-17A (35, 36). Such changes in the immune microenvironment bring additional antitumor benefits to patients, and in combination with immunotherapy may cause antitumor “immune superposition phenomenon”.

In advanced HNSCC, high-dose radiotherapy and uncontrolled tumor growth lead to mucocutaneous ulceration or tumor rupture, as well as iatrogenic pseudoaneurysms. This can lead to ulceration and bleeding of the oral, nasal or head and neck skin, which can be life-threatening and difficult to manage clinically (37–39). The decrease in arterial wall strength and increased fragility due to early antitumor therapy or tumor invasion increases surgical difficulty. Surgery is associated with a high mortality rate and carries a risk of adverse events such as hemiplegia and tumor rebleeding (40). As a rapid, safe and effective means of tumor hemostasis, TACE has been increasingly used in refractory hemorrhage of head and neck tumors (41, 42). For treatment of tumor combined with hemorrhage, the main purpose of embolization is to stop bleeding, which has little impact on the tumor, and has become the main cause of tumor rebleeding due to continued tumor growth. Therefore, when embolizing the tumor blood vessels and stopping bleeding, tumor necrosis and shrinkage can achieve better hemostasis and control the tumor at the same time, prolong survival and improve quality of life. In this study, DEB-TACE was used to treat HNSCC combined with bleeding, and the clinical success rate of hemostasis in 10 patients was 100%. There was no rebleeding during follow-up, which achieved a good hemostatic effect and was considered to be related to the efficient tumor-shrinking effect of DEB-TACE. We observed changes in quality of life before and after treatment. Overall quality of life was significantly improved at 1 month after treatment, and fatigue, dyspnea and insomnia were also significantly reduced, which may have been related to the effective control of tumors and reduction of tumor burden by DEB-TACE.

The choice of embolic material diameter in TACE is critical to improve efficacy and reduce embolic complications (43, 44). Granular embolic agents may be better because of their uniform size and morphology and elasticity, but the choice of particle size is affected by factors such as tumor size, location and blood supply. Due to the possible “dangerous anastomosis” of intracranial and extracranial communication between the blood vessels of the head and neck, complications such as cerebral infarction can occur when embolization is careless (45). Previous studies have suggested that the internal diameter of the material used for TACE embolization should be >300 μm to avoid ectopic injury (46). However, when the tumor blood vessels are embolized by particles with too large a diameter, only proximal embolization can be achieved, and the establishment of collateral circulation greatly reduces their efficacy (47, 48). The smaller the diameter of the embolization particles, the easier it is for them to be deposited in peripheral blood vessels, which should result in better in therapeutic efficacy. Smaller DEBs can be deposited further into the tumor and better loaded with extended-release drugs, effectively reducing the risk of embolism in non-targeted areas (49). Laurent et al. observed the distribution of microspheres after embolization of nasopharyngeal angiofibromas and paragangliomas by triacrylic gelatin microspheres. They concluded that compared with larger-diameter microspheres, the distribution of 100–300-μm microspheres was consistent in internal and external tumor vessels, and the risk of non-target embolization by small microspheres did not increase in the standardized embolization procedure (50). With the development of interventional medicine, especially the application of microcatheter super-selection arterial technology, non-target/shunt vessels can be avoided, so there is more choice for the inner diameter of embolic materials. In this study, microcatheter super-selection technology was used to select the proximal end of the tumor when performing TACE, and microspheres with a diameter of 100–300 μm were selected. The whole DEB-TACE process was carried out under fluoroscopic observation and strictly followed the technical requirements of DEB embolization. The patients in this study had no serious complications such as ectopic embolization. The main adverse reactions after surgery were pain and fever, which were mild and possibly related to tumor swelling and necrosis after embolization, and improved within a short period of time after medical treatment. We believe that 100–300-μm CalliSpheres are safe for embolization of tumor blood vessels in head and neck malignant tumors. The application of fine super-selection technology during surgery can avoid wedge catheter position and frequent angiography during single vessel embolization, which can reduce the risk of non-target embolization. In this study, the adverse reactions related to immunotherapy were mainly fatigue, hypothyroidism and rash; most of which were grade 1/2. Only three patients had grade 3 adverse reactions (2 cases of fatigue and 1 of rash), and no serious immune adverse effects occurred. All patients improved after symptomatic treatment, no new immunotherapy-related adverse events were observed in all patients during follow-up period, and DEB-TACE combined with PD-1 inhibitor showed good tolerability.

This study had some limitations. Firstly, it was a retrospective and non-randomized controlled study, which may have led to some bias. Secondly, meaningful subgroup analyses could not be performed because of the small sample size. Thirdly, the interventional procedure-performing physicians were not unified, so the correlation between super-selective catheterization from a greater distance for precise blockage and improved interventional effect could not be analyzed. Large-sample, prospective, randomized controlled clinical trials are needed to verify the efficacy and safety of this method. Also, the mechanism behind the positive results of this combined therapy requires further exploration.

This study preliminarily showed that CalliSpheres TACE combined with PD-1 inhibitor was safe and effective for treatment of unresectable HNSCC, significantly prolonged survival and improved quality of life, and provides a good treatment option for HNSCC.

## Data Availability

The original contributions presented in the study are included in the article/Supplementary Material. Further inquiries can be directed to the corresponding authors.
